# Effects of Heat Stress on the Ruminal Epithelial Barrier of Dairy Cows Revealed by Micromorphological Observation and Transcriptomic Analysis

**DOI:** 10.3389/fgene.2021.768209

**Published:** 2022-01-13

**Authors:** Zitai Guo, Shengtao Gao, Jun Ding, Junhao He, Lu Ma, Dengpan Bu

**Affiliations:** State Key Laboratory of Animal Nutrition, Institute of Animal Sciences, Chinese Academy of Agricultural Sciences, Beijing, China

**Keywords:** heat stress, dairy cow, ruminal epithelium, milk protein, rumen fermentation

## Abstract

Heat stress (HS) alters the rumen fermentation of dairy cows thereby affecting the metabolism of rumen papillae and thus the epithelial barrier function. The aim of the present study was to investigate if HS damages the barrier function of ruminal epithelia. Eight multiparous Holstein dairy cows with rumen cannula were randomly equally allocated to two replicates (*n* = 4), with each replicate being subjected to heat stress or thermal neutrality and pair-feeding in four environmental chambers. Micromorphological observation showed HS aggravated the shedding of the corneum and destroyed the physical barrier of the ruminal epithelium to a certain extent. Transcriptomics analysis of the rumen papillae revealed pathways associated with DNA replication and repair and amino acid metabolism were perturbated, the biological processes including sister chromatid segregation, etc. were up-regulated by HS, while the MAPK and NF-kB cell signaling pathways were downregulated. However, no heat stress-specific change in the expression of tight junction protein or TLR4 signaling was found, suggesting that HS negatively affected the physical barrier of the ruminal epithelium to some extent but did not break the ruminal epithelium. Heat stress invoked mechanisms to maintain the integrity of the rumen epithelial barrier by upregulating the expression of heat shock protein and repairments in rumen papillae. The increase in amino acid metabolism in rumen papillae might affect the nutrient utilization of the whole body. The findings of this study may inform future research to better understand how heat stress affects the physiology and productivity of lactating cows and the development of mitigation strategies.

## Introduction

Heat stress (HS) has been a major concern for dairy producers in tropical and subtropical areas, especially in summer, as HS can not only decrease milk yield but also decrease the content of milk protein (Gao et al., 2017; Guo et al., 2018). Recently, perturbation of the inflammatory response during HS was reported to be responsible, at least partially, for the declined milk protein synthesis (Gao et al., 2019). The host inflammatory response was shown to be related to some of the abnormal metabolites in the rumen, especially lipopolysaccharide (LPS) (Mani et al., 2012). In a previous study, we revealed that HS increased the concentration of volatile fatty acid (VFA) in the rumen fluid before feeding (Gao et al., 2017). The alterations of rumen fermentation in HS cows are similar to those observed in cows that suffer from subacute ruminal acidosis (SARA). Indeed, the decline of rumen pH during SARA increases the lysis of Gram-negative bacteria, resulting in a rapid increase in LPS (Danscher et al., 2015). However, it is difficult to determine the specific changes in rumen fermentation in cows under HS.

It has been shown that HS can increase the intestinal permeability of monogastric animals (Sakurada and Hales, 1998; Hall et al., 2001; Leon and Helwig, 2010). However, it is not known if HS also increases the permeability of the rumen epithelia, which are structured differently than the intestinal epithelia. Unlike the intestinal epithelia of single monolayer cells connected with tight junctions (Shen and Turner, 2006), the ruminal epithelia have a multi-layered structure of stratified squamous epithelium, the granulosum that has tight junctions, spinosum, and basale (Graham and Simmons, 2005). The complex structure of the rumen papillae also plays an important role in defending against harmful substances in rumen fluid, however, the blood diversion from the viscera to the periphery might alter the ruminal epithelial morphology under heat stress (Kregel, 2002; Lambert et al., 2002). Though a recent study showed that mild HS did not induce barrier dysfunction of the rumen papillae in lactating dairy cows probably owing to a defense mechanism and feeding adaptation (Eslamizad et al., 2020). The damages of HS to the barrier function of ruminal epithelia in lactating dairy cows remain elusive. We hypothesized that HS might damage the barrier function of ruminal epithelia and induce tissue inflammation, which could eventually decrease milk protein synthesis. To this end, we evaluated the effect of HS on the micromorphology and gene expression of the rumen epithelia in lactating cows.

## Materials and Methods

### Animals and Study Design

Eight multiparous Holstein dairy cows (238 ± 10 DIM; 618 ± 100 kg of BW; 23 ± 2.8 kg of milk/d) each with a permanent rumen cannula were used in the current study. Due to environmental chamber availability, the study was carried out in two replicates with four different cows in each replicate as reported in a previous study (Sun et al., 2019). When four cows were being used in a replicate experiment, the other cows were kept in a free-stall barn cooled with running fans until they were required for another replicate experiment. The four cows in one replicate were randomly allocated to four individual environmental chambers (Beijing Kooland Technologies Co., Ltd.) that had 12 h light (0600–1800) and 12 h dark (1800–0600) cycles. For the first 7 days, all cows were maintained at thermal neutral conditions (20°C and 55% relative humidity) and fed *ad libitum* for adaptation. The experiment period lasted for 18 days including 9 days of the control phase and 9 days of the trial phase. In the control phase, all cows continued to be in thermal neutral conditions [20°C and 55% of RH as configuration; 0600–1800 h of light] and fed *ad libitum*. While in the trial phase, two of the four cows were exposed to cyclical heat stress conditions (HS, 0600–1,800 h at 36°C, 1,800 to 0600 h at 32°C, and 40% of RH) and fed *ad libitum*, whereas the other two cows were maintained at the same thermal neutral conditions as mentioned above but pair-fed (PFTN). The amount of feed provided to the PFTN cows was calculated based on the average feed intake of the HS cows 1 day earlier as previously described by Wheelock et al. (2010); thus the trial for the PFTN cows started 1 day after the HS cows. All the cows had free access to drink water and were fed a total mixed ration (TMR) formulated to meet the predicted requirements of NRC in energy, protein, minerals, and vitamins (Table 1). The cows were individually fed twice a day (0500 and 1,700 h). The cows were milked twice a day (0500 and 1700 h) and the milk yield was recorded at each milking time. After the previous four cows exited the chamber, the next four cows were randomly allocated to the four chambers to repeat the experiment as described above. In total, HS and PFTN each had four cows (*n* = 4).

**TABLE 1:** Ingredients and nutrients of experimental diet (DM basis).

Item	Value
Ingredients (% of DM)	
Bean meal	10.42
Cotton meal	5.03
Rapeseed meal	2.18
DDGS^a^	5.45
Feeding corn meal^b^	1.15
Steam-flaked corn	23.98
Limestone	0.91
Salt	0.55
Magnesium Oxide	0.36
Dicalcium Phosphate	0.42
Fat powder	1.15
Sodium bicarbonate	0.97
Supplement^c^	0.67
Corn silage	28.77
Alfalfa hay	17.99
Chemical analysis (% of DM)	
NDF^d^	27.69
ADF^e^	18.57
CP^f^	15.31
Ash	7.88
Organic matter	92.12
Ether extract	2.1
NE_L_ ^g^ (Mcal/kg of DM)	1.69

a Distillers dried grains with solubles.

b Flour made with corn.

c Contained (per kg of DM) a minimum of 250,000 IU, of vitamin A; 65,000 IU, of vitamin D; 2,100 IU, of vitamin E; 400 mg of Fe; 540 mg of Cu; 2,100 mg of Zn; 560 mg of Mn; 15 mg of Se; 35 mg of I; and 68 mg of Co.

d Neutral detergent fiber.

e Acid detergent fiber.

f Crude protein.

g Net energy of lactation.

### Sampling and Measurements

In both the control and HS trial phases, rectal temperature (RT), skin temperature (ST), and respiratory rate (RR) were recorded for each cow four times daily (0100, 0700, 1,300, and 1900 h). To calculate the precise temperature-humidity-index (THI) inside the chamber, ambient temperature (AT) and RH were recorded four times daily (0100, 0700, 1,300, 1900 h) automatically with an electronic thermometer; THI was calculated using the equation below as previously described (Buffington et al., 1981).

The weight of orts from each cow was recorded daily before the morning feeding. On d 2, 4, 6, and 8 of the control and HS trial phases, samples of rumen fluid were collected *via* rumen cannula before morning feeding. The pH of the rumen fluid was immediately measured after sampling using an electronic pH meter. Then the rumen fluid samples were filtered through four layers of gauze and divided into two aliquots (10 ml each), with one aliquot being acidified with 0.1 ml of 6 M HCl for ammonia concentration determination, while the other aliquot being preserved following the addition of 1 ml of 25% metaphosphoric acid for analysis of VFA. All samples of rumen fluid were stored at −20°C until analysis. Milk samples were collected daily from morning and afternoon milking (25 ml for each milking) and mixed equally and stored at 4°C until analysis after bronopol tablet (D&F Control System, San Ramon, CA) was added as a preservative.

Before morning feeding on d 9 of the trial phase in each replicate, six samples of rumen papillae were collected using forceps *via* the rumen cannula from each cow. Three papillae samples were gently rinsed in 0.9% NaCl solution as described by Dieho et al. (2016), immediately frozen in liquid nitrogen, and then stored at −80°C until RNA isolation, while the other three papillae samples were fixed in buffered 4% paraformaldehyde for micromorphological observation.

The fixed papillae samples were rinsed and dehydrated in a series of ethanol baths and then deparaffinized in xylene. Each papillae sample was stained with hematoxylin and eosin (H&E) and then observed under a Leica S9 Stereo microscope (Leica Microsystems Inc., Buffalo Grove, United States) as described previously (Nishihara et al., 2019).

### Analysis

Feed samples were dried at 65°C for 48 h and ground with a Wiley mill (Arthur H. Thomas Co., Philadelphia, PA) for analysis of ash, DM, CP, NDF, and ADF content. The NDF content was measured using a fiber analyzer (Ankom Technology, A200, Macedon, NY) using the method of Van Soest et al. (1991), but α-amylase and sodium sulfide were used. The Ash, DM, CP, and ADF contents were analyzed according to the AOAC Official Method (Horwitz et al., 2010, 942.05 for ash; 2001.12-2005 for dry matter; 988.05 for CP, and 973.18 for ADF).

Milk samples were analyzed with the method of infrared spectrophotometry using an automatic analyzer of milk composition (MilkoScan Type 78,110, Foss Electric, Hillerød, Denmark). The concentration of VFA in rumen fluid samples was determined using gas chromatography (Agilent 6890A, Agilent Technologies, California, United States) as described by Hu et al. (2005). Ammonia-N in the rumen fluid samples was measured as described by Broderick and Kang (Broderick and Kang, 1980).

Specimens of rumen papillae were microscopically examined and analyzed using a panoramic scanner (3DHISTECH Ltd., H-1141 Budapest, Hungary), meanwhile the results were assembled and viewed. For each specimen, four clear areas were selected and used to measure the total thickness of ruminal epithelia and the thickness of the corneum and granulosum. To acquire the distance, a segment perpendicular to the screen section was made and measured *via* the ruler tool in the application.

The effect of the HS on the gene expression in papillae was evaluated using transcriptomics. The RNA extraction and sequencing, analysis of differently expressed genes (DEGs) and Go and KEGG enrichment analysis were conducted following our former research (Gao et al., 2021), during which the Dynamic Impact Approach (DIA), Database for Annotation, Visualization, and Integrated Discovery (DAVID) were used.

### Statistical Analysis

Data of lactation performance, rumen fermentation, parameters of rumen papillae, and vital signs were statistically analyzed using SAS v. 9.4 (SAS Institute, Cary, NC) with all data tested for normality. All data were analyzed using PROC MIXED. Significance was declared at *p* ≤ 0.05, and the tendency was declared at 0.05 < *p* ≤ 0.10.

Analysis of differently expressed genes (DEGs) was conducted with the DESeq2 package (1.26.0) in R (3.6.1). The mapped read count tables of individual samples and genes were used as the standard workflow instructed. The false discovery rate (FDR) obtained by the method of Benjamin and Hochberg was used to correct the *p-*value. Genes with *p* < 0.05 were regarded as DEGs.

## Results

### The Effect of HS on Vital Signs, Lactation Performance, and Rumen Fermentation of Dairy Cows

The effects of HS on vital signs, lactation performance, and rumen fermentation are reported in Table 2. In brief, HS increased the RR, RT, and ST (2.65-fold, 1.33°C and 4.59°C, respectively; *p* < 0.01) compared with the PFTN conditions, and reduced milk yield (by 34.22%, *p <* 0.05) and the content of milk protein (by 10.44%, *p* < 0.05) compared with the PFTN conditions. In addition, HS increased the concentration of total VFA (by 31.60%, *p* < 0.01), acetate (by 39.51%, *p* < 0.05), propionate (by 47.29%, *p* < 0.05), and valerate (by 61.54%, *p* < 0.05) in rumen fluid, while decreased the rumen liquor pH compared with PFTN. The HS cows tended to have increased concentration of butyrate (by 20.35%, 0.05 < *p* < 0.1) but not isobutyrate or isovalerate.

**TABLE 2:** The vital signs, lactation performance and rumen fermentation of dairy cows.

Items	PFTN^a^	HS	SEM	*p*-value	
				Treatment	Period
THI^b^	68.92	83.11	0.7996	0.0002	0.0145
RR^c^ (counts/min)	27.42	72.62	3.7667	0.0006	0.4904
RT^d^, °C	38.49	39.82	0.4505	0.0484	0.3095
ST^e^, °C	32.30	36.89	0.2374	0.0002	0.3138
DMI^f^, kg/d	10.25	10.01	1.0783	0.8293	0.1206
Milk yield, kg/d	17.94	11.80	2.2121	0.0480	0.2112
Protein, %	3.64	3.26	0.1387	0.0436	0.6024
Fat, %	4.88	5.41	0.9828	0.6295	0.8379
Lactose, %	4.74	4.92	0.5317	0.7408	0.7902
SCS^g^	7.31	8.40	1.8059	0.5783	0.0199
pH	6.71	6.34	—	0.0335	0.5840
LPS^h^, EU/mL *10^5^	0.1817	0.1861	0.0285	0.8780	0.5218
NH_3_-N, mg/dL	17.04	19.07	2.2663	0.4707	0.2407
Total VFA^i^	58.01	76.34	2.3998	0.0001	0.9284
Acetate, mmol/L	32.65	45.55	4.2921	0.0189	0.7827
Propionate, mmol/L	14.25	20.99	1.9927	0.0244	0.9488
Isobutyrate, mmol/L	0.68	0.58	0.0579	0.1875	0.6024
Butyrate, mmol/L	7.96	9.58	0.8364	0.0671	0.9911
Isovalerate, mmol/L	1.29	1.15	0.1585	0.4265	0.9848
Valerate, mmol/L	0.65	1.05	0.0676	0.0107	0.6748

a Pair-feeding thermal neutral.

b Temperature-humidity index.

c Respiration rate.

d Rectal temperature.

e Skin temperature.

f Dry matter intake.

g Somatic cell score.

h Lipopolysaccharide.

i Volatile fatty acid.

### The Effect of HS on Rumen Papillae

**FIGURE 1:**
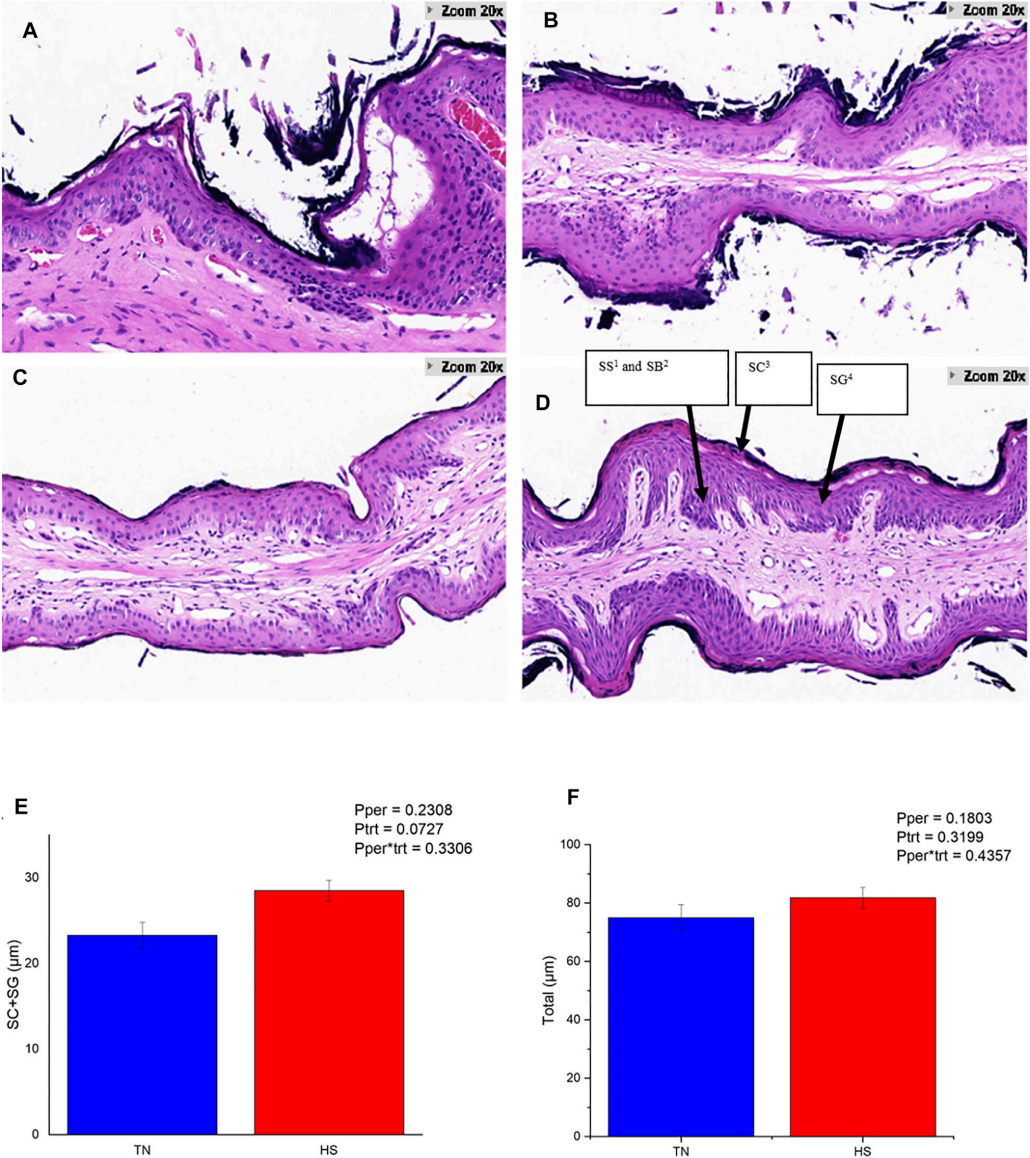
The rumen papillae structure of HS cows **(A,B)** and PFTN cows **(C,D)**, and their thickness of the stratum corneum (SC) plus stratum granulosum (SG) layers of the rumen epithelium **(E)** as well as total epithelial thickness **(F)**. 1) Stratum Spinosum.2) Stratum Basale.3) Stratum Corneum.4) Stratum Granulosum.

The HS cows showed obvious damage and slough off of the corneum (Figures 1A,B), while the PFTN cows had intact corneum (Figures 1C,D). Heat stress enlarged the intercellular space of the granulosum and spinosum and induced obvious separations of layers inside the rumen papillae (Figure 1B). Furthermore, HS tended to increase the thickness of corneum and granulosum (23.20 vs. 28.39 μm; 0.05 < *p* < 0.1; Figure 1E) but did not affect the thickness of the whole rumen papillae (Figure 1F).

### The Effect of HS on the Gene Expression in Rumen Papillae

**FIGURE 2:**
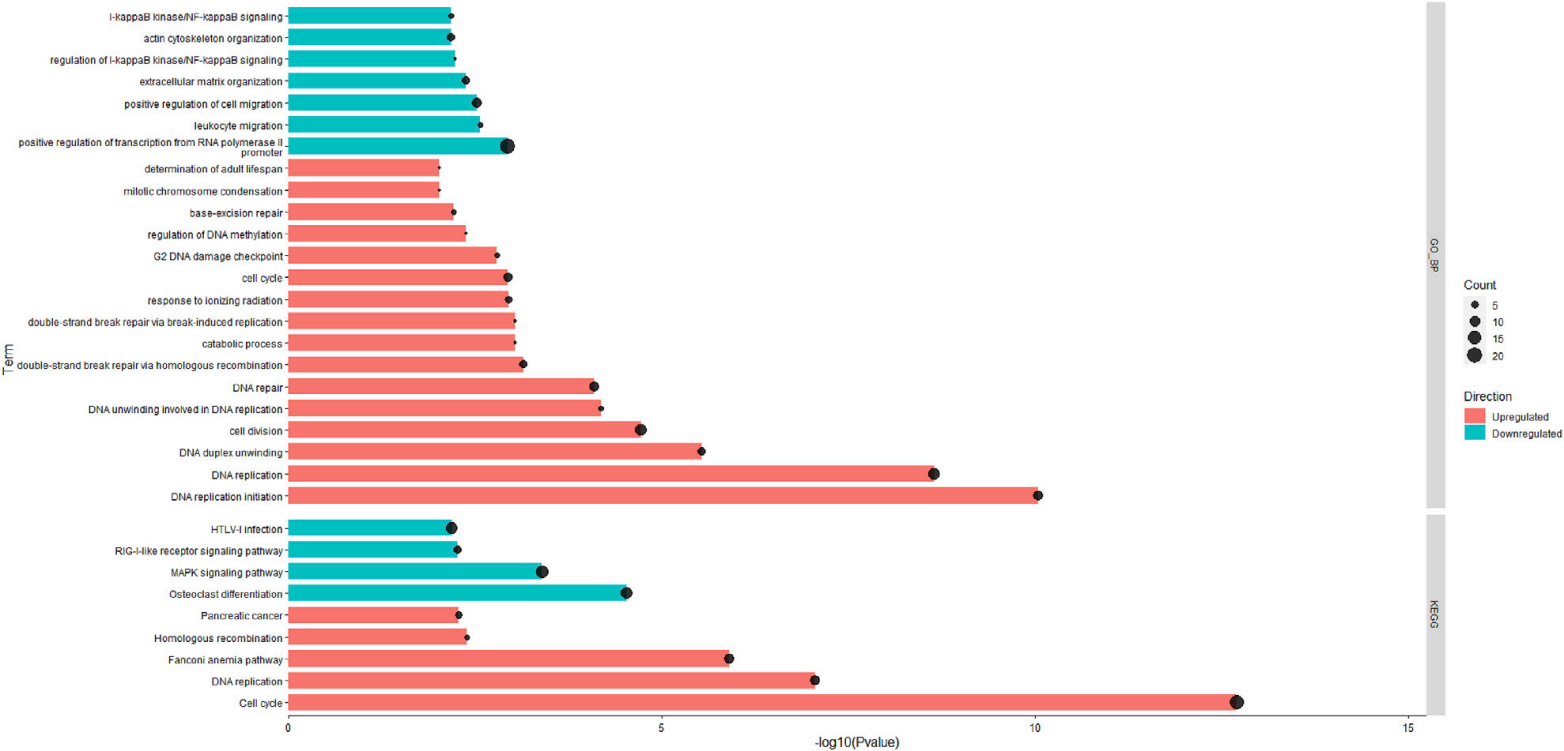
The significantly enriched pathways in rumen epithelial tissues of the HS and the PFTN cows as determined using Database for Annotation, Visualization and Integrated Discovery (DAVID) analysis against the GO_BP and KEGG databases.

A total of 15,654 unique expressed genes were detected in the tissue of rumen papillae. Between the HS and PFTN cows, 501 genes were DEGs, including 238 up-regulated and 263 down-regulated (Padj ≤0.05). The methods of DIA and DAVID were used in the functional analysis of the DEGs. The whole DIA output, the results of perturbations on the main categories and subcategories of the KEGG in rumen papillae between HS and PFTN cows, and the top 20 most impacted pathways, as uncovered by the DIA, in the rumen papillae of HS cows compared with PFTN cows are available in supplement File S2. All the results of DAVID analysis using the KEGG and GO Biological Process (GO_BP) are shown in Figure 2. In the HS cows, the GO_BP analysis revealed 7 and 16 different terms that were downregulated DEGs and upregulated DEGs (*p* ≤ 0.05), respectively.

## Discussion

Heat stress significantly decreases feed intake and milk performance (Beede and Collier, 1986; West, 2003; Ranjitkar et al., 2020). A THI of 68–69 was considered to cause some HS when evaluated with respect to rising respiratory rates and rectal temperature (Brügemann et al., 2012; Collier et al., 2012; Umar et al., 2021). However, in this study, the HS cows suffered a more severe reduction in milk yield compared with PFTN cows, which is consistent with our previous study (Gao et al., 2017). The results of vital signs, milk yield, and THI indicated that the HS and pair-fed model was successfully performed. Evidently, HS probably directly decreased milk synthesis through the regulation of the growth hormone axis as proposed previously (Rhoads et al., 2010).

As the major products of rumen fermentation, VFA are the main energy source for ruminants (Sutton et al., 1988; Owens and Basalan, 2016). However, slight perturbations in rumen fermentation can change the concentration of individual VFAs. Heat stress can increase the frequency of feed ingestion and drinking (Eslamizad et al., 2020; Herbut et al., 2021). It is believed that such adaptations were the results of self-regulation in response to HS (West, 2003). Robles et al. (2007) found that increasing the frequency of daily feeding (2 or 4 times) increased the concentration of total VFA compared with cows fed only once daily. In the current study, the amount of ingested fermentable organic matter was similar between the HS and the PFTN cows. There was a small chance that feeding changes would affect VFA concentration by reducing or increasing DMI. Faster dilution in the rumen caused by increased water intake was proved to promote microbial growth in an early study (Nocek and Braund, 1985). The rumen microbes can provide moderate concentrations of fermentation end product source of the ingesta provided by the ruminant (Calamari et al., 2013), which were ultimately metabolized to VFAs and absorbed through the rumen wall (Hyder et al., 2017). Thus, the higher rumen liquid turnover rate in the HS cows caused by feeding changes might be attributable to their increased VFA concentration in the rumen.

Elevated concentrations of propionate and butyrate in the rumen can stimulate the development of the rumen papillae, hence enhancing VFA absorption across the ruminal epithelium (Kristensen and Harmon, 2004; Storm et al., 2011). Butyrate is likely to be oxidized into β-hydroxybutyrate after being absorbed in the ruminal epithelium cells, directly providing energy to the rumen papillae (Ślusarczyk et al., 2010). Steele et al. (2012) showed that butyrate promotes the length of rumen papillae by perfusion trials. Moreover, a low concentration of butyrate was shown to inhibit cell apoptosis (Xu et al., 2018). Therefore, elevated rumen concentrations of propionate and butyrate corroborate the increased thickness of the corneum and granulosum observed in the HS cows.

The corneum of rumen papillae in the HS cows was shed and the inside layers appeared separated. The corneum is in direct contact with rumen content and is colonized with rumen microorganisms and thus the epithelial tissues are updated regularly (Lavker and Sun, 1983). The update frequency is related to diet ingredients, with high-concentrate diets significantly reducing the renewal frequency. Tajima et al. (2001) indicated that long-term feeding of high-concentrate diets decreased the acetate:propionate ratio in the rumen fluid whereas it increased the concentration of total VFA, suggesting that ruminal corneum exfoliation in HS cows may also be attributed to increased total rumen VFA concentration. In addition, the transport and absorption of VFA by the ruminal epithelium depend on the integrity of the corneum and the degree of keratinization (Galfi et al., 1991; Del Bianco Benedeti et al., 2018). Yohe et al. (2019) reported that the exfoliation of the corneum and the hyperkeratosis of the epithelium reduce the ability of the ruminal epithelium to transport and absorb VFA. Therefore, the increased VFA concentration in the HS cows might be a major reason for the exfoliation of the corneum.

The HS treatment affected the expression of some genes in the rumen papillae. Among the 20 most impacted pathways (as detected with the DIA analysis), HS activated one pathway related to membrane transport (ABC Transporter). The ABC transporters transport various substrates including amino acids, peptides, and cellular metabolites across the cell membrane (Schneider and Hunke, 1998; Dean et al., 2001). Except for the upregulation of ABC transporters, HS upregulated the expression of HSPA5 and DNAJB9, which encode heat shock proteins (Hsp) 70 and 40, respectively. Hsp has functions in maintaining physiological and stabilizing the structure of cells (Sharp et al., 1999), and among all the known Hsp, Hsp70 has the most prominent functions in most animals under stress (Kiang and Tsokos, 1998; Bhat et al., 2016). Considerable increases in the expression of Hsp are usually directly associated with stress (Feder and Hofmann, 1999). Herein, the upregulation of Hsp observed in the rumen papillae of the HS cows is beneficial to the repair and maintenance of the cells. As a result, the absorption and utilization of amino acids in the rumen papillae increased as indicated by the upregulation of six amino acid metabolism pathways by HS. The up-regulation of the amino acids metabolism indicates that amino acid utilization in the ruminal epithelium was enhanced by Hsp in the HS cows, suggesting the amino acid concentrations in rumen fluid were increased. The source of amino acids in rumen fluid was mainly from diet protein as well as the degradation of rumen microorganisms. Some rumen microorganisms could directly synthesize some amino acids from VFA and ammonia. The increased rumen concentration of VFA could provide the carbon source for amino acid synthesis, meanwhile causing the death of gram-negative bacteria in rumen. Furthermore, the concentrations of the amino acids whose metabolism was up-regulated by HS were also increased in the rumen by SARA, and SARA can also exacerbate the breakdown of bacteria and increase the rumen concentration of amino acids. Thus, the activation of amino acids metabolism in the papillae in the HS cows might be similar to that observed in cows suffering from SARA, which is mostly related to the changing of rumen fermentation.

Besides up-regulating the metabolism of amino acids, HS also influenced the replication of DNA and the repair of ruminal epithelial cells. Four of the 20 most impacted pathways are relevant to the replication and repair of genetic information processing, among which homologous recombination, Fanconi anemia pathway is related to the repair process (San Filippo et al., 2008; Krokan and Bjørås, 2013; Rodríguez and D’Andrea, 2017). While homologous repair is the most important mechanism of repair of DNA double-strand breaks (Baumann and West, 1998). HS leads to excessive production of reactive oxygen species (ROS) in dairy cows, inducing oxidative stress (OS) in their body (Guo et al., 2021). The mitochondrial dysfunction following OS can induce pro-apoptotic factors in the mitochondrial inner membrane and activate endogenous apoptosis and ultimately lead to tissue apoptosis (Kannan and Jain, 2000; Antonsson, 2001). Propionate and butyrate were believed to play an important role in cell growth, but several studies have contradictory opinions on the effects of these two VFAs (Baldwin, 1999; Shen et al., 2005). Liu et al. (2014) indicated that butyrate needed to be used in combination with insulin-like growth factor-1 (IGF-1) to significantly promote DNA replication, and otherwise it can inhibit DNA replication. In the present study, we found up-regulation of the gene encoding insulin-like growth factor binding protein 5 in the HS cows, which might enhance the biological effects of IGF-1. Therefore, the OS induced by HS could aggravate the damage to the epithelial cells in rumen papillae. Such damage might activate the repairing of intracellular DNA. To protect the epithelial cells, Hsp synthesis needs to increase, and ruminal epithelial cells need to upregulate their amino acid metabolism. Because nutrients are partitioned between the liver and mammary gland in cows under HS (Bu et al., 2017), the increase in amino acid metabolism in rumen papillae would decrease the supply of precursors for milk production by the mammary glands. However, further correlation analysis of nutrient utilization among the rumen epithelia, mammary gland, and other tissues is still needed to support this premise.

The results of our DAVID analysis verified the activation of replication and repair in rumen papillae. All the up-regulated DEGs are involved in DNA replication and cell division, while the down-regulated DEGs are involved in the MAPK signaling pathway and the NF-kB cell signaling pathway in rumen papillae. Mitogen-activated protein kinases (MAPKs) are a type of protein kinases that are widely present in animal cells. They are activated in cells in which DNA damage or oxidative damage occurs, inducing a pro-apoptotic effect (Seger and Krebs, 1995; Zumsande and Gross, 2010). The TLR4/NF-kB pathway is closely related to the anti-inflammatory immunity of the body (Zusso et al., 2019). Moreover, one study has found that TLR4 in epithelial cells could recognize LPS and activate related pathways (Bäckhed et al., 2002). Taken together, the rumen papillae in the HS cows did not exhibit leukocyte migration or up-regulation of the NF-kB pathway, suggesting that TLR4 related upstream receptors might have not been activated. Furthermore, the expressions of tight junction protein including claudin, occludin, and ZO-1 showed no difference between the HS and PFTN cows. The tight junctions among ruminal epithelial cells are the most important connection between cells and serve as the barrier preventing harmful substances from the rumen (Zhang et al., 2019; Ma et al., 2021). They can also assist in the transport of ions and nutrients. The results of the present study suggest that the expression of Hsp may participate in the protection of tight junctions.

## Conclusion

Heat stress exhibited direct impacts on rumen fermentation and metabolism of rumen papillae. Heat stress promoted the proliferation of the rumen papillae but aggravated the shedding of the corneum and may negatively affect the physical barrier of the ruminal epithelium to a certain extent. The increase in VFA concentration induced by heat stress might stimulate the development of rumen papillae. However, heat stress did not break the intact barrier function, since neither a change in tight junctions nor perturbation to inflammatory response was observed in ruminal papillae, and heat stress did not alter the expression of TLR4-related upstream receptors. Heat stress up-regulated the expression of heat shock protein and activated the repair of damaged epithelial cells in rumen papillae. These mechanisms contribute to the maintenance of the integrity of rumen tissue. The up-regulated metabolism of amino acids along with Hsp synthesis may affect the supply of the precursors for milk protein synthesis, but correlation analysis of the utilization of amino acids in organs and the whole body is needed in future studies.

## Data Availability

The original contributions presented in the study are publicly available. This data can be found here: https://www.ncbi.nlm.nih.gov/sra/?term=PRJNA778529. The supplementary material are available online at https://doi.org/10.6084/m9.figshare.17041181, File S1: Dataset of differently expressed genes; File S2: The output of DIA analysis; File S3: The output of DAVID analysis.
